# Stimulated phosphorylation of ERK in mouse kidney mesangial cells is dependent upon expression of Cav3.1

**DOI:** 10.1186/s12882-022-02844-1

**Published:** 2022-06-16

**Authors:** Sudha Priya Soundara Pandi, Michael J. Shattock, Bruce M. Hendry, Claire C. Sharpe

**Affiliations:** 1 Department of Inflammation Biology, King’s College London, Denmark Hill Campus, James Black Centre, London, SE5 9NU UK; 2 Clinical and Experimental Sciences, Faculty of Medicine, University of Southampton, Southampton, UK; 3 School of Cardiovascular Medicine and Sciences, King’s College London, London, UK

**Keywords:** Calcium channels, CRISPR-cas9, Mesangial cell, ERK1/2, Glomerulonephritis

## Abstract

**Background:**

T-type calcium channels (TTCC) are low voltage activated channels that are widely expressed in the heart, smooth muscle and neurons. They are known to impact on cell cycle progression in cancer and smooth muscle cells and more recently, have been implicated in rat and human mesangial cell proliferation. The aim of this study was to investigate the roles of the different isoforms of TTCC in mouse mesangial cells to establish which may be the best therapeutic target for treating mesangioproliferative kidney diseases.

**Methods:**

In this study, we generated single and double knockout (SKO and DKO) clones of the TTCC isoforms Ca_V_3.1 and Ca_V_3.2 in mouse mesangial cells using CRISPR-cas9 gene editing. The downstream signals linked to this channel activity were studied by ERK1/2 phosphorylation assays in serum, PDGF and TGF-β1 stimulated cells. We also examined their proliferative responses in the presence of the TTCC inhibitors mibefradil and TH1177.

**Results:**

We demonstrate a complete loss of ERK1/2 phosphorylation in response to multiple stimuli (serum, PDGF, TGF-β1) in Ca_V_3.1 SKO clone, whereas the Ca_V_3.2 SKO clone retained these phospho-ERK1/2 responses. Stimulated cell proliferation was not profoundly impacted in either SKO clone and both clones remained sensitive to non-selective TTCC blockers, suggesting a role for more than one TTCC isoform in cell cycle progression. Deletion of both the isoforms resulted in cell death.

**Conclusion:**

This study confirms that TTCC are expressed in mouse mesangial cells and that they play a role in cell proliferation. Whereas the Ca_V_3.1 isoform is required for stimulated phosphorylation of ERK1/2, the Ca _V_3.2 isoform is not. Our data also suggest that neither isoform is necessary for cell proliferation and that the anti-proliferative effects of mibefradil and TH1177 are not isoform-specific. These findings are consistent with data from in vivo rat mesangial proliferation Thy1 models and support the future use of genetic mouse models to test the therapeutic actions of TTCC inhibitors.

**Supplementary Information:**

The online version contains supplementary material available at 10.1186/s12882-022-02844-1.

## Background

Mesangial cells (MCs) in the glomeruli of the kidney are essential in maintaining the structural integrity of the glomerular capillary network [1]. In healthy adult kidneys, MCs remain in a quiescent state with a cell renewal rate of < 1% per day, as shown by autoradiography [2]. With various stress stimuli, including increases in blood pressure and blood sugar, MCs show aberrant proliferation. The uncontrolled MC proliferation can induce extracellular matrix (ECM) accumulation in the glomeruli, leading to glomerulosclerosis [3–5]. These features are recognised as a critical element in the pathogenesis of chronic kidney disease (CKD) secondary to many glomerular diseases, including diabetic nephropathy and IgA nephropathy.

The proliferation of MC is tightly regulated by various mitogenic stimuli including platelet derived growth factor (PDGF), PI3/AKT and tyrosine kinases [6–9]. In addition to this, the influx of Ca^2+^ via calcium channels appears to be involved in the regulation of MC proliferation. Alongside the different types of high voltage-gated calcium channels including P/Q type, N-type, R-type, and L-type channels are the low voltage-gated T-type calcium channels (TTCC) [10]. It has been recognised for decades that calcium channel blockers can impact MC proliferation through mechanisms beyond L-type inhibition [11]. The specific target of these actions is uncertain, though they are unlikely to be P/Q-type, N-type or R-type calcium channels as there is no evidence of their expression in MC. TTCC on the other hand, are expressed in human and rat MC and are sensitive to inhibition by the TTCC blocker TH1177 in vitro and in vivo in the Thy1 nephritis rat model [12, 13]. Also, the knockdown of TTCC in human and rat MC has been shown to inhibit proliferation [12, 14]. TTCC exhibit a similar structure to that of LTCC but differ in kinetics and activation threshold [10]. The blockade of TTCC also exhibits anti-proliferative properties in vascular and pulmonary arterial smooth muscle cells [15, 16] and human cancer cells [17]. In aortic smooth muscle cells, T-type calcium currents are predominantly present in the G1 phase and the synthesis phase of the cell cycle [15]. There are three isoforms of TTCC including Cav3.1(α1_G_), Ca_V_3.2(α1_H_) and Ca_V_3.3(α1_I_) [10]. To date, the role of specific TTCC isoforms in MC proliferation is not well studied. Additionally, there are no known reports available that show the presence of TTCC in mouse MC.

As no selective and specific small molecule inhibitors of TTCC isoforms exist, we aimed to investigate the expressions of TTCC isoforms in mouse MC in vitro and the impact of deletion of specific TTCC isoforms on cell proliferation and phosphorylation of ERK1/2, a key process in the Ras-MAPK pathway controlling cell-cycle progression [18]. We have, therefore, developed single and double knockouts (SKO and DKO) of Ca_V_3.1 and Ca_V_3.2 by using the clustered regularly interspaced short palindromic repeats (CRISPR)/ CRISPR associated protein 9 (Cas9) gene editing system. We have also examined the stimulated phosphorylation of ERK1/2 in Ca_V_3.1 and Ca_V_3.2 SKO MC clones as compared to WT cells and evaluated proliferation of Ca_V_3.1 and Ca_V_3.2 SKO MC with and without non-selective TTCC blockers (mibefradil and TH1177).

## Methods

### Cell culture

Mouse mesangial cells (SV40 MES13) purchased from ATCC (Virginia, United States) were maintained in a 3:1 solution of Dulbecco’s modified Eagle’s medium (DMEM) (Invitrogen, Paisley, UK) and F-12 (Invitrogen). The medium was supplemented with 5% fetal bovine serum (FBS), 14 mM HEPES, 100 IU/ml penicillin, 100 μg/ml streptomycin, and 2.5 μg/ml amphotericin (Invitrogen). Cultured cells were maintained at standard cell culture condition at 37 °C with 95% O_2_ and 5% CO_2_.

### Calcium channel inhibitors

Mibefradil (Sigma Aldrich, Dorset, UK) and verapamil (Sigma Aldrich) were made from 10 mM stock solutions in distilled water and stored at 4 °C, and every two months, fresh stock solutions were prepared. TH1177 was a kind gift from Dr Lloyd Gray, University of Virginia, Charlottesville, Va., USA. TH1177 was prepared from a 10 mM stock solution in 100% ethanol and stored at -20 °C.

### Serum, PDGF and TGF-β1 stimulation

MES13 cells were seeded at 3 × 10^5^ cells in 35 mm dishes with 1% FBS for 48 h. At 48 h, cells were cultured in varied conditions: 0% FBS, 20% FBS, or 20 ng/ml PDGF-BB and 10 ng/ml TGF-β1 (R&D Systems, Minneapolis, MN) and vehicle control [TGF- β1 buffer-4 mM HCl plus 0.1% BSA, pH3] for 24, 48 and 72 h time points. Cells were harvested for RNA extraction at the end of each time point. The medium was removed and washed with PBS and 350 µl of RLT buffer with beta-mercaptoethanol was used to lyse the cells. The RNA extraction was carried out using the RNeasy Mini Kit (Qiagen Ltd, Crawley, UK) by following the manufacturer’s instructions.

### Reverse transcriptase- polymerase chain reaction (RT-PCR)

cDNA was synthesised from 2 µg of RNA using high capacity RNA to cDNA synthesis kit (Applied biosystems, Massachusetts, USA) in the 20 µl reaction mix. RT-PCR amplification was carried out from 5 µl of cDNA using Dream Taq polymerase (Fischer Scientific, Loughborough, UK) using a thermocycler. The PCR amplification was carried out with an initial denaturation of 95 °C for 3 min, 35 cycles of denaturation 95 °C for 30 s, annealing Tm-5 for 30 s, extension 72 °C for 1 min and final extension 72 °C for 5 min. The RT-PCR primers are listed in Suppl. Table S1.

### CRISPR-cas9 plasmids

plenti-CRISPR-cas9-gRNA for Ca_V_3.1/Ca_V_3.2 was purchased from GenScirpt, New Jersey, USA. The plasmid contained a plentiCRISPRv2.0 backbone cloned with cas9, ampicillin, bleomycin, puromycin selection markers, and 20 nucleotides of the gRNA sequence complementary to exon 4 of Cacna1G and exon 6 of Cacna1H gene under the control of U6 promoter (Suppl. Fig. S2a, c and b).

### Transfection, antibiotic and clonal selection

plenti-CRISPR-cas9-gRNA for Ca_V_3.1/Ca_V_3.2 plasmids were used to generate stable knockouts which were transfected into MES13 cells using lipofectamine 3000 (Thermo fisher Scientific, Massachusetts, USA). Briefly, 6 × 10^5^ cells were seeded in 6 well plates overnight in the antibiotic-free growth medium. The following day, for SKO generation, 5 µg of plenti-CRISPR-cas9-gRNA for Ca_V_3.1 or Ca_V_3.2 plasmids and for DKO generation, 2.5ug each of plenti-CRISPR-cas9-gRNA for Ca_V_3.1 and Ca_V_3.2 were transfected by lipofection, and the medium was changed after 6 h. Post 72 h of transfection, complete growth medium with 2 µg/ml of puromycin was added for antibiotic selection. The cells resistant to puromycin post 72 h antibiotic selection were trypsinised and sub-cultured to the new six-well plate and the second round of antibiotic selection was carried out using puromycin to minimise the contamination of untransfected cells. Although the cells were sorted with a selection marker, there remained a possibility of a mixed population of cells with a variable length of base-pair deletion of ~ 1–19 nucleotides. Hence, the knockout cells were subjected to single-cell clonal selection by serial dilution starting with 500 cells in 96-well plates. The SKO clones were picked, expanded and confirmed at the genomic level by Sanger sequencing and confirmed at the translational level by Western blot. This second round of puromycin selection resulted in too few cells to achieve single cell clonal selection for the DKO clones and expansion for Western blotting, hence DKO cells from the first round of antibiotic selection went through clonal selection in the conditional medium. The survived clones were sent for Sanger sequencing alone.

### Genomic DNA extraction, PCR and Sanger sequencing

MES13 control cells and Ca_V_3.1, Ca_V_3.2 SKO and DKO clones were subjected to genomic DNA extraction by using the QIAamp DNA extraction kit (Qiagen Ltd, Crawley, UK), following the manufacturer’s instructions. The genomic DNA was quantified in a Nanodrop, and 100 ng of DNA was amplified by using Dream Taq polymerase and primers spanning the gRNA sequence region in 20 µl reaction mix. The primer list is detailed in Suppl. Table S1. The PCR products were cleaned using the ExoSAP-IT Express PCR product clean-up kit (Thermo Fisher Scientific). The PCR product was preceded to Sanger sequencing by GATC Biotech, Ebersberg, Germany.

### MTS assay

MES13 cells and SKO clones of Ca_V_3.1 and Ca_V_3.2 treated with or without calcium channel blockers (mibefradil and TH1177) were subjected to a microculture tetrazolium (MTS) assay (Promega, Southampton, UK) to measure the cell number. Briefly, MES13 cells, Ca_V_3.1 and Ca_V_3.2 SKO clones were subjected to serum deprivation in 1% FBS for 48 h. Cells were seeded into 96-well plates at a density of 5000 cells per well and incubated with varied concentration of mibefradil and TH1177 (0, 5, 10 and 20 µM) in 5%FBS. Absorbance at 492 nm was measured at 48, and 72 h in a microplate reader.

### Signal transduction evaluation

MES13 and SKO clones of Ca_V_3.1 and Ca_V_3.2 were seeded at 6X10^5^ cells per well in 6 well plates and left overnight. The cells were serum deprived to 1% FBS for 48 h. At 48 h, the cells were stimulated for 30 min with either: 5% FBS, 20 ng/ml PDGF + 1% FBS or 10 ng/ml TGF-β1 + 1% FBS.

Similarly, MES13 cells alone were seeded at 6X10^5^ cells per well in 6 well plates and left overnight. The cells were serum deprived to 1% FBS for 48 h. At 44 h, the cells were treated with 5 µM mibefradil + 1% FBS and at 48 h, the cells were stimulated with 5% FBS, 20 ng/ml PDGF + 1%FBS and 10 ng/ml of TGF- β1 + 1%FBS for 30 min.

The medium was removed immediately after 30 min stimulation, washed with PBS, and the cells were lysed with RIPA buffer with protease and phosphatase inhibitor (Sigma). The lysate was scraped off from the plates and incubated on ice for 30 min with intermittent vortexing. The lysates were centrifuged for 14,000 rpm at 4 °C for 20 min. The supernatant from the lysate was transferred to new labelled tubes and stored at -80 °C. The experiments were repeated three times.

### Western blot

The protein concentration was quantified by BCA assay (ThermoFisher Scientific), and 20–40 µg of protein was separated in 8% PAGE gel. The proteins were transferred to a nitrocellulose membrane at 35 V for 3 h for Ca_V_3.1 and Ca_V_3.2 and 100 V for 1 h for pERK1/2. The protein transfer was confirmed by Ponceau staining, and the blots were washed and incubated with 5% non-fat milk (Sigma) for 1 h. The blots were incubated with primary antibodies: Ca_V_3.1 (1:1000) (Alomone, Jerusalem, Israel) and Ca_V_3.2 (1:500) (Alomone), pERK1/2 (1:5000) (Cell signaling, London, UK), total ERK1/2 (1:5000) (cell signaling) and beta-actin (1:5000) (Abcam, Cambridge, UK) overnight at 4 °C. The following day, the blots were washed thrice with 1 × TBS + 0.1% Tween 20 (TBS-T) for 10 min and then incubated with anti-rabbit secondary antibody conjugated with horse radish peroxidase for 1 h at room temperature. The blots were washed thrice with 1 × TBS-T and incubated with ECL substrate (Fisher scientific) for 5 min and exposed to X-ray film. In order to probe for total ERK1/2 and beta-actin, the blots were stripped with stripping buffer (Sigma) for 30 min at RT under dark. Protein band density was quantified using Image Studio Lite software.

### Statistics

All data were tested for normality and no difference in the variances between groups was detected using the Shapiro–Wilk test. Parametric variables were analysed using a One-Way Analysis of Variance (for multiple group comparisons) with a post hoc Bonferroni test or Two-Way Analysis of Variance with post hoc Tukey test *p* < 0.05 is considered as statistical significance. Statistical analyses were performed using Prism software version 8.0 (Graph-Pad Software, San Diego, CA).

## Results

### Pharmacological T-type calcium channel inhibition is anti-proliferative in wild-type mouse mesangial cells

**Fig. 1:**
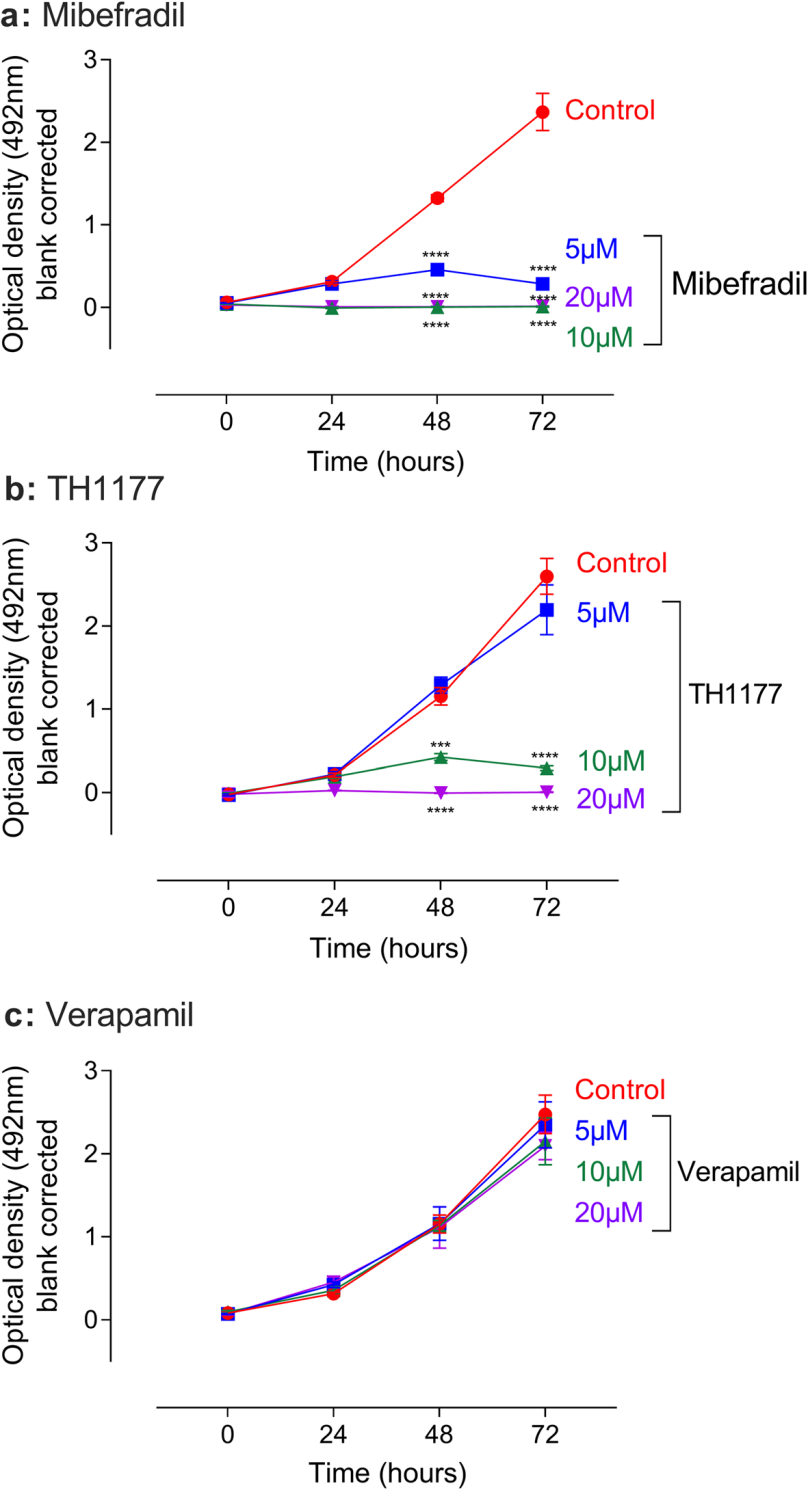
Modulation of Mouse mesangial cell proliferation by TTCC blockers. MES13 cells treated with TTCC blockers mibefradil (**a**), TH1177 (**b**) from 0–20 µM concentration shows dose-dependent inhibition of mesangial cell proliferation in comparison to vehicle control and L type calcium channel blocker verapamil (**c**). The data are represented as mean ± SD. *n* = 4, ***P < 0.001, ****P < 0.0001 is the comparison between the concentration to vehicle control. Two-Way ANOVA with Turkey post hoc correction

The expression of Cav3.1, Cav3.2 and Cav 3.3 in wild type (WT) mouse MC (MES13) was confirmed by reverse transcriptase PCR followed by agarose gel electrophoresis (Suppl. Fig. S1). Figure 1 shows that there was a dose-dependent inhibition of MC cell proliferation with TTCC inhibitors mibefradil (Fig. 1a) and TH1177 (Fig. 1b) with an apparent ED50 of 5–10 µM. This was in contrast to no inhibition of cell proliferation with the LTCC blocker verapamil at up to 20 µM (Fig. 1c). These data are consistent with published work on human and rat MC [12, 14] and confirm a role for TTCC but not LTCC in mouse MC stimulated proliferation.

### Blocking of T-type calcium channels in mouse mesangial cells inhibits the phosphorylation of ERK1/2

**Fig. 2:**
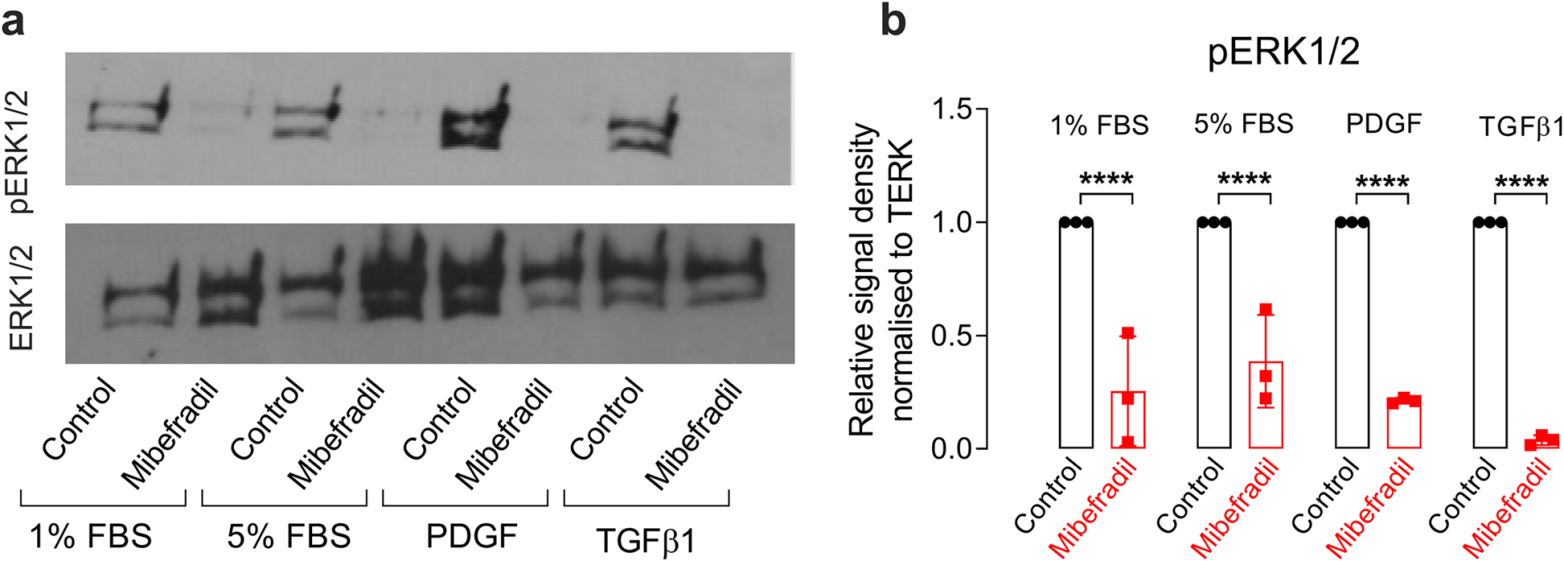
Mibefradil inhibits phosphorylation of ERK1/2 in the presence of 5%, FBS, PDGF or TGFβ1. A representative Western blot showing phosphorylated ERK1/2 levels in MES13 cells with and without mibefradil treatment in 1% FBS (**a**) for 4 h, in all media conditions (with 5% FBS, PDGF or TGFβ1). The full-length images of Western blot are presented in Supplementary figure S5. (**b**) shows the mean densitometry quantification of pERK1/2 levels (normalised to total ERK1/2) in three independent experiments with MES13 cells treated with mibefradil for 4 h and controls in a range of media conditions (5% FBS, PDGF or TGFβ1). The data are represented as mean ± SD, *n* = 3, ****p < 0.0001, from a One-Way ANOVA with Bonferroni post hoc correction

**Fig. 3:**
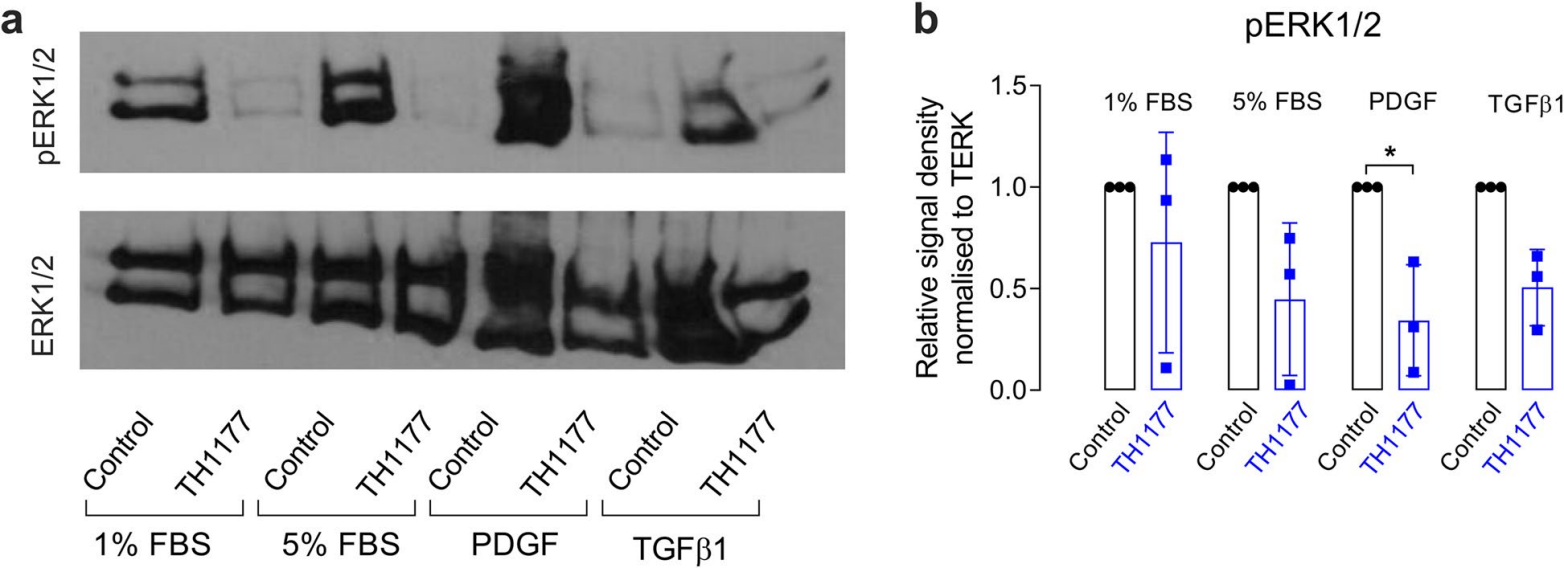
TH1177 inhibits phosphorylation of ERK1/2 in the presence of 5%, FBS, PDGF or TGFβ1. **a** shows a representative Western blot showing phosphorylated ERK1/2 levels in MES13 cells with and without TH1177 treatment for 4 h, in all media conditions (with 5% FBS, PDGF or TGFβ1). The full-length images of Western blot are presented in Supplementary figure S6. **b** shows the mean densitometry quantification of pERK1/2 levels (normalised to total ERK1/2) in three independent experiments of MES13 cells treated with TH1177 for 4 h and controls in a range of media conditions (5% FBS, PDGF or TGFβ1). The data are represented as mean ± SD, *n* = 3, * *p* < 0.05, from a One-Way ANOVA with Bonferroni post hoc correction

MAPK signaling plays a major role in regulating mesangial cell proliferation [19, 20]. Hence stimulated phosphorylation of ERK1/2 was evaluated in the WT control cells (MES13) treated with 5% FBS, PDGF or transforming growth factor-beta 1 (TGF-β1) with or without TTCC blockers mibefradil and TH1177. Figure 2 shows that stimulated phosphorylation of ERK1/2 is clearly reduced by mibefradil. TH1177 also inhibits the pERK1/2 response in 5% FBS, TGF-β1 and PDGF (Fig. 3).

### Ca_V_3.1 and Ca_V_3.2 deletion in mouse mesangial cells by CRISPR-Cas9 gene editing

CRISPR-cas9-Ca_V_3.1/ Ca_V_3.2 transfection with single-cell clonal selection allowed us to obtain cells with Ca_V_3.1, Ca_V_3.2 SKO as determined by PCR and Western blot. There was no compensatory change seen in the other TTCC isoform in each case. Details of the methodology and verification of knockdown are shown in the Supplementary Material and Supplementary Figures S2, S3 and S4. DKO cells fail to survive, hence only sequencing was performed to confirm the knockout, suggesting knockout of both the TTCC isoforms is lethal to cell survival. This also suggests that expression of at least one of the two isoforms is needed for cell proliferation.

### Knockout of Ca_V_3.1, not Ca_V_3.2 inhibits the phosphorylation of pERK1/2

**Fig. 4:**
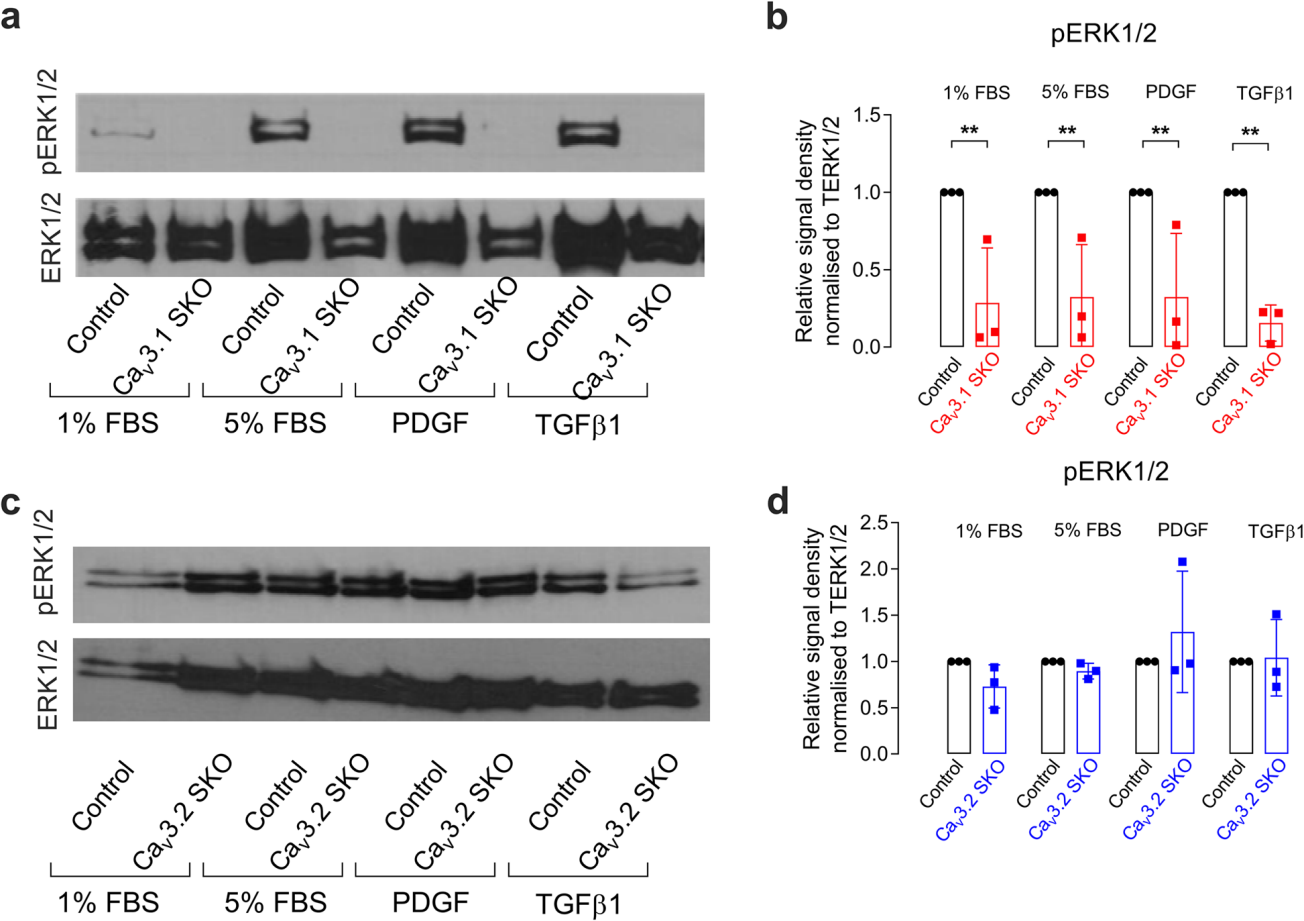
Ca_V_3.1 but not Ca_V_3.2 SKO in 5% FBS, PDGF and TGF-β1inhibits phosphorylation of ERK1/2. A representative Western blot showing phosphorylated ERK1/2 levels in MES13 control cells, Ca_V_3.1 SKO clone (**a**) and Ca_V_3.2 SKO clone (**c**) in all media conditions (with 5% FBS, PDGF or TGF-β1). The full-length images of Western blot are presented in Supplementary figure S7. **b** shows the mean densitometry quantification of pERK1/2 levels (normalised to total ERK1/2) in three independent experiments of Ca_V_3.1 SKO clones and control in a range of medium conditions (5%FBS, PDGF or TGF-β1). The data are represented as mean ± SD, *n* = 3, *p < 0.05, **p < 0.01, ***p < 0.001, from a One-way Anova with Bonferroni post hoc correction. **d** shows similar densitometry for CaV3.2 KO cells

To evaluate the role of the different TTCC isoforms Ca_V_3.1 and Ca_V_3.2 on the activation of the MAPK pathway, the SKO clones were treated with 5% FBS, PDGF or TGF-β1 and pERK1/2 *versus* total ERK1/2 assayed by Western blot. The level of stimulated pERK1/2 protein in the Ca_V_3.1 SKO clone was significantly reduced in 1% FBS, 5% FBS, PDGF and TGF-β1 compared to WT control cells (Fig. 4a and b). Conversely, the level of stimulated pERK1/2 in the Ca_V_3.2 SKO clone was the same as in WT control cells in all the treatment conditions (Fig. 4c and d). This suggests that the activation of the pERK1/2 by a variety of pro-proliferative stimuli is mediated via Cav3.1 with Cav3.2 playing little or no role.

### Knockout of Ca_V_3.1 or Ca_V_3.2 results in a significant but small reduction in cell proliferation

The Ca_V_3.1 and Ca_V_3.2 SKO clone were used to evaluate the biological significance of these TTCC isoforms in cell proliferation. We evaluated the effect of the TTCC blockers on the stimulated proliferation of Ca_V_3.1 and Ca_V_3.2 SKO clones as compared to WT cells.

**Fig. 5:**
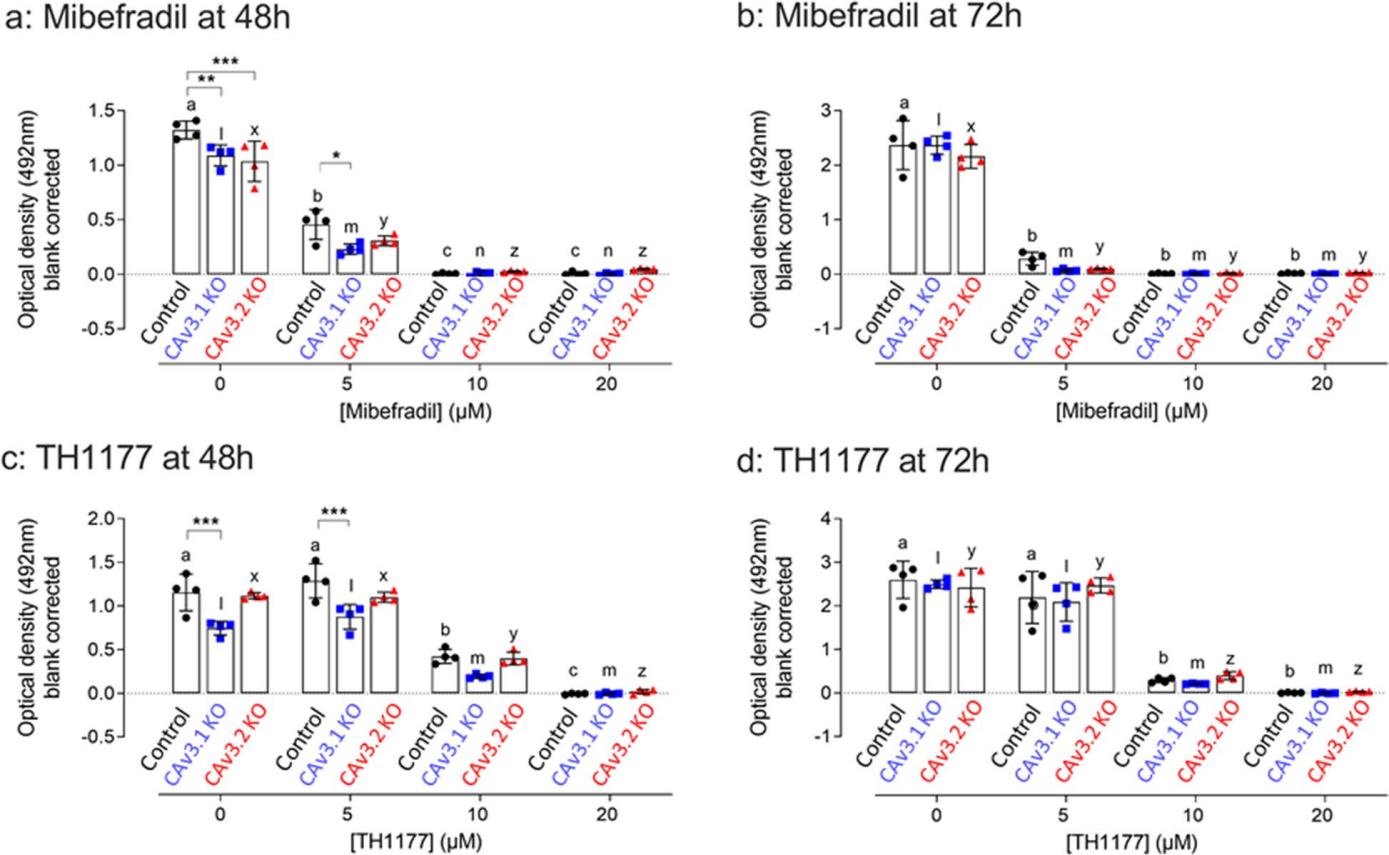
Modulation of Ca_V_3.1 SKO, Ca_V_3.2 SKO and MES13 cell proliferation by TTCC blockers. Cell proliferation of the Ca_V_3.1, Ca_V_3.2 knockout cells after treatment with TTCC blockers: mibefradil (**a, b**) and TH1177 (**c, d**) were compared to wild type control cells (MES13). Cell number was calculated by using the MTS assay in time points at 48 and 72 h. The data are represented as mean ± SD. Letters ‘abc’, ‘lmn’ and ‘xyz’ are used to denote the significance between drug concentration in control, Ca_V_3.1 and Ca_V_3.2 respectively. The same letter above the graphs indicates no change between the groups. Different letters above the graph indicate the significant difference between the means. The data are represented as mean ± SD, *n* = 4, **P < 0.01, ***P < 0.001 are for the comparison between KO to its respective control at each concentration. Two-Way ANOVA with Turkey post hoc correction

In the absence of TTCC blockade, both Ca_V_3.1 and Ca_V_3.2 SKO clones exhibited a small but significant reduction in serum-stimulated proliferation at 48 h compared to WT control cells (Fig. 5a and c). These differences were not apparent at 72 h (Fig. 5b and d). Both the Ca_V_3.1 and Ca_V_3.2 SKO clones remained sensitive to inhibition of proliferation by mibefradil and TH1177 as seen in Fig. 5, where the apparent ED50 for inhibition is broadly similar in the WT control and SKO cells. There was a tendency for the Ca_V_3.1 SKO clone to show reduced proliferation in the presence of low doses TTCC inhibitor compared with WT cells.

## Discussion

In this study, we have demonstrated that i) mouse MC express TTCC and are sensitive to two TTCC inhibitors: mibefradil and TH1177. However, MC are insensitive to the LTCC inhibitor, verapamil; ii) The TTCC isoforms Ca_V_3.1 and Ca_V_3.2 were successfully knocked out in mouse MC by CRISPR-cas9 gene editing, creating viable single-cell TTCC isoform SKO clones; iii) Ca_V_3.1 SKO, but not Ca_V_3.2 SKO inhibits the serum, PDGF and TGF- β1 stimulated phosphorylation of ERK1/2; iv) The effect of Ca_V_3.1 SKO on pERK1/2 is similar to the actions of TTCC blockers (mibefradil and TH1177) in WT cells; v) Knock out of either Ca_V_3.1 or Ca_V_3.2 alone does not alter the anti-proliferative effects of mibefradil or TH1177 and has minimal effects on stimulated cell proliferation, whereas knock out of both isoforms completely inhibits proliferation.

TTCC and their role in MC proliferation have been reported in a few studies. Findings in rat and human MC have shown that only Ca_V_3.1 and Ca_V_3.2 were detected in rat MC and Ca_V_3.2 is expressed in human MC [12–14]. However, in our study, we have shown that all three TTCC isoforms (Ca_V_3.1, Ca_V_3.2 and Ca_V_3.3) were expressed in mouse MC. Various studies have used mibefradil and TH1177 as an inhibitor of TTCC in proximal tubular epithelial cells, MC and other cell types. Mibefradil has a 10- fold selectivity to TTCC than LTCC [21, 22], whereas TH1177 may inhibit other ion channels. In prostate cancer cells, TH1177 inhibits proliferation by impeding the entry of Ca^2+^, but it does not affect calcium release from internal stores [23]. TH1177 is reported to affect a range of cation channels including TRPV5 channels [24]. Studies in rat and human MC show that the MC were sensitive to the TTCC inhibitors mibefradil and TH1177 but there was no effect seen by the LTCC inhibitor (verapamil) [12, 14]. Similar sensitivity was shown in our mouse MC to mibefradil and TH1177 with no effect of verapamil. As TTCC blockers can be non-selective, to accurately understand the role of TTCC, it is important to look at the effect of the different isoforms in signaling and proliferation.

The role of CaV3.1 in cell proliferation has been described in heart, lung and cancer cells [15, 25–28]. Human pulmonary artery myocytes express CaV3.1 and silencing of CaV3.1 inhibits serum-induced proliferation [25]. This is consistent with studies in preadipocytes in primary culture. The level of CaV3.1 in preadipocytes is high and is downregulated during differentiation. Knockdown of CaV3.1 or use of mibefradil inhibits preadipocyte proliferation [29]. CaV3.1 also plays a different role in the cancer cells. Knockdown of CaV3.1 indeed induces cell proliferation and reduces apoptosis in MCF-7 breast cancer cells. The effect was reversed by overexpression of CaV3.1. The effect of CaV3.1 knockdown is similar to the TTCC blocker (tarantula toxin ProTx-1). In MCF-7 cells, knockdown of CaV3.2 did not affect proliferation or apoptosis [28]. All these studies used siRNA to transiently silence the CaV3.1 or CaV3.2 isoforms and evaluate their roles in proliferation. There have been no studies to date demonstrating the specific role of TTCC isoform on the MC proliferation in the kidney. TTCC inhibition experiments in rat and human MC also did not delineate the specific role of TTCC isoforms, though we have previously shown that although Cav3.2 is upregulated in the cortex of kidneys with Thy1 nephritis, this is not in the glomerular compartment. Cav3.1 is predominantly expressed in glomeruli, though this does not increase with disease [12]. We have therefore addressed the specific mesangial roles of Cav3.1 and Cav3.2 in this study using CRISPR-cas9 gene editing. In our study, mouse MCs lacking either Ca_V_3.1 or Ca_V_3.2 survived and formed single-cell clones whereas Ca_V_3.1 and Ca_V_3.2 DKO clones didn’t survive to carry out the western blot. This suggests that expression of either one of the TTCC isoforms is sufficient to support cell proliferation and cell survival.

Given the wealth of evidence suggesting a role for T-type Ca channels in cell proliferation, it is surprising that knocking out each isoform individually has virtually no impact on cell proliferation. However, since the relationship between T-type Ca current and proliferation is extremely steep (see Fig. 4 in [30]), with only a small residual current being permissive for proliferation, it seems that the presence of either isoform is sufficient to allow effectively normal proliferation. The absence of both isoforms however is lethal—presumably by completely preventing cell cycle progression. On the other hand, pharmacological inhibition, with drugs showing little of no isoform selectivity, allows a graded reduction in Ca influx and hence a graded anti-proliferative effect.

MC proliferation is controlled by various regulators of the cell cycle, including PI3/AKT, Ras/MAPK, and calmodulin-dependent kinase signaling [8, 9]. These are likely to be involved in the changes seen in MC proliferation in TTCC KO clones. MAPK plays an important role in mesangioproliferative disease and inhibition of Ras/ERK1/2 by a pharmacological inhibitor or Ras antagonist reduces glomerular cell proliferation [19, 20]. It has been shown that in rat pulmonary artery smooth muscle cells (PASMCs) stimulation with insulin growth factor -1 (IGF-1) induces Ca_V_3.1 expression, and the level was decreased when treated with both PI3K and MEK inhibitor [15]. PASMCs treated with constitutively active AKT increase basal Ca_V_3.1 expression more than with constitutively active MEK treatment, suggesting upregulation of Ca_V_3.1 is via AKT signaling [15]. Similarly, Ca_V_3.1 is highly expressed in prostate cancer tissue. Knockdown of Ca_V_3.1 in PC-3 and Dol45 cells suppressed cell proliferation by inhibiting the transition of cells to G1/S phase. The Ca_V_3.1 knockdown in the cancer cells decreases phosphorylated AKT (pAKT) protein which was recovered by ectopic AKT expression [27]. Furthermore, idiopathic pulmonary arterial hypertension cells (iPAH) are more proliferative than cells from control pulmonary arteries. TTCC blocker TTA-A2 reduced the proliferation of iPAH cells by delaying the S/G2 transition. TTA-A2 induces pAKT but not pERK1/2 or phosphorylated P38, suggesting the proliferation of iPAH is diverted to AKT signaling [26]. All these studies suggest that the Ca_V_3.1-dependent regulation of proliferation is mostly via AKT signalling.

The present results in mouse MC reveal a significant role for Ca_V_3.1 in the stimulated phosphorylation of ERK1/2 with no such role for Ca_V_3.2. The effects of both Cav3.1 and 3.2 SKO on MC proliferation were much more subtle. Ca_V_3.1 SKO cells without pERK1/2 responses were slightly but not profoundly hypoproliferative. This may be explained by compensatory changes in the SKO cells to amplify pERK1/2-independent proliferative signaling. Similarly, TTCC inhibitors were effective in reducing proliferation in Ca_V_3.1 SKO cells that already had impaired pERK1/2 responses. This implies that the pERK1/2 pathway actions of these agents may not be the mechanism of their anti-proliferative actions. TTCC activity may be coupled to proliferation by multiple pathways. Moreover, these small molecule TTCC inhibitors likely have a range of actions on cation channels, including inhibition of TRP channels [24]. These off-target effects are another possible explanation for their anti-proliferative actions.

## Conclusion

Treatment with the non-selective TTCC inhibitors, mibefradil and TH1177 or deletion of Ca_V_3.1 in mouse MC prevents the stimulated phosphorylation of ERK1/2 but deletion of Ca_V_3.2 does not. Deletion of either isoform alone has a limited impact on cell proliferation whereas treatment with mibefradil and TH1177 is highly anti-proliferative. Deletion of both Ca_V_3.1 and Ca_V_3.2 results complete inhibition of cell proliferation and cell death. This is consistent with a previous report from our group showing reduced pERK1/2 immuno-localisation in the glomeruli of TH1177-treated animals with Thy1 nephritis [12]. This study justifies further work using genetic models in mice to further delineate the roles of TTCC in mesangial cell biology and the pathology of mesangioproliferative CKD. Use of conditional and cell-specific KO should allow these ideas to be tested. Isoform-selective TTCC inhibitors may also help to dissect the actions seen and warrant further study as potential therapeutics in mesangioproliferative glomerular disease.

## Supplementary Information

**Additional file 1:** **Supplementary material**. Bacterial transformation, plasmidpurification and validation, selection and validationof single-cell clones. **SupplementaryFigure S1. **Confirmation of TTCC isoform in Mouse mesangial cells andmodulation of its proliferation by TTCC blockers. (a, b, c) RT-PCR for the Ttype calcium isoforms were detected in mouse mesangial cells (MES13). The bandin each lane in the agarose gel represent the PCR product of the T type calciumisoforms Cacna1G, Cacna1H and Cacna1I (PCR product size: CaV3.1-376; CaV3.2-594;CaV3.3-528). mRNA of the CaV3.3 in 72 h treatment with PDGF and TGFβ1 aloneshown faint band. **Supplementary FigureS2. **Validation of CRISPR-cas9- gRNA for cacna1G and Cacna1H. plenti-CRISPR-cas9-cacna1G a) andplenti-CRISPR-cas9-cacna1H b) vector map expressing gRNA mapping to exon 4 of36 exons containing cacna1G gene and on exon 6 of 35 exons containing cacna1Hgene under the control of U6 promoter, with selection markers ampicillin forprokaryotic and puromycin for mammalian cell selection. a) and b) plasmids wereconfirmed by restriction digestion by MluI restriction enzyme and by sangersequencing using primer designed for U6 promoter. (gRNA sequences:Cacnca1G-TCCGTGTGCTGCGACCGCTC, Cacna1H- ACCTGACGAA GGCGCTGTCC). Gel: Panel A:Lane 1-10KB ladder, Lane 2 –circular plasmid, Lane 3-No restriction enzymecontrol, Lane 4- plasmid digested by Mlu. Panel B: Lane 1 –circular plasmid,Lane 2-No restriction enzyme control, Lane 3- plasmid digested by Mlu, Lane4-10KB ladder. Yellow highlight - gRNA sequence. **Supplementary Figure S3. **Confirmation of Ca_V_3.1 and Ca_V_3.2knockout at the genomic level. a) PCR amplificationfor Ca_V_3.1 (left panel) and Ca_V_3.2 (right panel) genes inthe six clones each from cacna1G and cacna1h by using primers spanning gRNA region. Cacna1G clone 2 and Cacna1H clone 3harbouring indel mutation shows PCR product shift compare to control. b)Sequencing analysis of cacna1G and cacna1H knockout in the clone 2 of cacna1G,clone 3 of cacna1H. Sequencing resultsshow deletion of the gRNA sequence with the frameshift mutation compare to wildtype (WT) control, confirming the knockouts. Right panel-sequencing forcacna1G; left panel- sequencing for cacna1H. Yellow highlight represents the sequencebefore gRNA in the gene and grey highlight represent the gRNA sequence. **Supplementary Figure S4. **Confirmation of cacna1G and cacna1H knockout at thepost-translational level. Western blot for Ca_V_3.1(a) protein expression in the single-cell clones of Ca_V_3.1 knockoutshow absence of bands in all six Ca_V_3.1 KO clones compared to wildtype cells and the positive control mouse brain. (b) single-cell clone of Ca_V_3.2KO shows the absence of bands in 5 clones compared to wild type and positivecontrol HEK cell overexpressing Ca_V_3.2. Loading control- actin. **Supplementary FigureS5: **Figure shows the full-length image ofWestern blot for Figure 2a described in the main text. **Supplementary Figure S6: **Figure shows the full-length image ofWestern blot for Figure 3a described in the main text. **Supplementary Figure S7: **Figure shows the full-length image ofWestern blot for Figure 4a and 4c described in the main text. **Supplementary Table S1: **Primers list, 1.28 MB, PDF. 

## Data Availability

Data from this study will be available from the corresponding author on reasonable request.

## Abbreviations

MC: Mesangial cell
TTCC: T-type calcium channel
LTCC: L- type calcium channel
CRISPR: Clustered regularly interspaced short palindromic repeats
cas9: CRISPR associated protein 9
KO: Knockout
ECM: Extracellular matrix
CKD: Chronic kidney disease
IgA: Immunoglobulin A
FBS: Fetal bovine serum
PDGF: Platelet derived growth factor
TGF-β1: Transforming growth factor – beta 1
RNA: Ribonucleic acid
DNA: Deoxyribonucleic acid
cDNA: Complementary deoxyribonucleic acid
RT-PCR: Reverse transcriptase- Polymerase chain reaction
ANOVA: Analysis of variance
