# Supplementary material for: Stimulated phosphorylation of ERK in mouse kidney mesangial cells is dependent upon expression of Cav3.1
Source: BMC Nephrol. 2022 Jun 16;23:211. doi: 10.1186/s12882-022-02844-1 (PMC9205043; doi:10.1186/s12882-022-02844-1)
Supplement: Supplementary file 1 — Additional file 1: Supplementary material. Bacterial transformation, plasmidpurification and validation, selection and validationof single-cell clones. SupplementaryFigure S1. Confirmation of TTCC isoform in Mouse mesangial cells andmodulation of its proliferation by TTCC blockers. (a, b, c) RT-PCR for the Ttype calcium isoforms were detected in mouse mesangial cells (MES13). The bandin each lane in the agarose gel represent the PCR product of the T type calciumisoforms Cacna1G, Cacna1H and Cacna1I (PCR product size: CaV3.1-376; CaV3.2-594;CaV3.3-528). mRNA of the CaV3.3 in 72 h treatment with PDGF and TGFβ1 aloneshown faint band. Supplementary FigureS2. Validation of CRISPR-cas9- gRNA for cacna1G and Cacna1H. plenti-CRISPR-cas9-cacna1G a) andplenti-CRISPR-cas9-cacna1H b) vector map expressing gRNA mapping to exon 4 of36 exons containing cacna1G gene and on exon 6 of 35 exons containing cacna1Hgene under the control of U6 promoter, with selection markers ampicillin forprokaryotic and puromycin for mammalian cell selection. a) and b) plasmids wereconfirmed by restriction digestion by MluI restriction enzyme and by sangersequencing using primer designed for U6 promoter. (gRNA sequences:Cacnca1G-TCCGTGTGCTGCGACCGCTC, Cacna1H- ACCTGACGAA GGCGCTGTCC). Gel: Panel A:Lane 1-10KB ladder, Lane 2 –circular plasmid, Lane 3-No restriction enzymecontrol, Lane 4- plasmid digested by Mlu. Panel B: Lane 1 –circular plasmid,Lane 2-No restriction enzyme control, Lane 3- plasmid digested by Mlu, Lane4-10KB ladder. Yellow highlight - gRNA sequence. Supplementary Figure S3. Confirmation of CaV3.1 and CaV3.2knockout at the genomic level. a) PCR amplificationfor CaV3.1 (left panel) and CaV3.2 (right panel) genes inthe six clones each from cacna1G and cacna1h by using primers spanning gRNA region. Cacna1G clone 2 and Cacna1H clone 3harbouring indel mutation shows PCR product shift compare to control. b)Sequencing analysis of cacna1G and cacna1H knockout in the clone 2 of cacna1G,clone 3 of [file 12882_2022_2844_MOESM1_ESM.pdf]

Supplementary material

Bacterial transformation, plasmid purification and validation

NEB 10-beta Competent Escherichia coli (high efficiency) was used for the transformation of plenti-CRISPR-cas9-gRNA for $Ca_V3.1$/ $Ca_V3.2$ plasmids. These plasmids at the concentration of 200 ng were mixed with the competent E.coli and incubated on ice for 30 min followed by heat shock at 42°C for 30 s. After 5 min of incubation in ice, 950 µl of SOC medium was added and incubated at 37°C shaker for 60 min. The transformed bacteria with the plasmids were spread onto the warm antibiotic selection plate (LB agar +100ug/ml ampicillin) and incubated overnight at 37°C. Single colonies of bacteria expressing plasmids were isolated and inoculated in 5 ml of LB broth+100 ug/ml ampicillin and incubated in 37°C shaker. After 6 h, 100 µl from the 5 ml culture was mixed with 25 ml of LB broth +100 ug/ml ampicillin for overnight at 37°C shaker. Plasmid purification was carried out from 25 ml bacterial culture expressing the plasmid using a Qiagen plasmid midi prep kit (Qiagen Ltd, Crawley, UK) by following the manufacturer's instructions. The purified plasmid DNA was quantified using a Nanodrop and validated by restriction enzyme digestion by incubating 1µg of the plasmid with 10,000 units of MluI restriction enzyme(New England Biolabs, Herts, UK) at 37°C for an hour. Restriction digestion was confirmed by running the digest on a 1% agarose gel against 10 KB ladder (Suppl. Fig. S2a-b). The purified plasmids were also validated by Sanger sequencing with U6-5' GGACTATCATATGCTTACCG primers (Suppl. Fig. S2a-b).

Selection and validation of single-cell clones

Around 20 single-cell clones each from Cav3.1 and Cav3.2 SKO were picked and six clones from each KO were selected and expanded for the further analysis. Whereas, only 3 clones from DKO were obtained. Six clones from each of the Cav3.1 and Cav3.2 SKO and 3 DKO clones were PCR amplified by using primers spanning the gRNA region and sent for Sanger sequencing. Out of 12 clones, clone 2 from Cav3.1, clone 3 and 6 from Cav3.2 displayed the pronounced KO effect as reflected in agarose gel (Suppl. Fig. S3a). It may be due to deletions of more than ten base pairs which are visible by the shift in the band compared to the control sample, whereas the rest of the clones did not show the shift. Hence, Sanger sequencing is essential to evaluate these clones, which can pick up even single base pair deletion. Sanger sequencing results confirmed that in comparison to control, 5 out of 6 clones of Cav3.1 KO and all six clones of Cav3.2 KO exhibited the deletion of few base-pairs. The clones of Cav3.2 knockout sequenced for Cav3.1 and Cav3.1 KO clone sequenced for Cav3.2 showed no base pair deletion, and its sequence pattern is similar to the control (Suppl. Fig. S3b). From this result, it confirmed that the KO of Cav3.1 and Cav3.2 does not have any impact on its counterpart's isoform. Sanger sequencing result of all 3 clones of DKO showed few pair deletion on both Cav3.1 and Cav3.2, confirming DKO (Suppl. Fig. S3c).

Though the sequencing result confirms the base pair deletion, the KO needs to be confirmed at the protein level. All six clones each from Cav3.1 KO and Cav3.2 KO were subjected to Western blot for Cav3.1 and Cav3.2 respectively. Similar to the sequencing result, there is a complete loss of Cav3.1 protein in all Cav3.1 clone except clone 5, which shows a faint band in Western blot in comparison to controls (WT control, clone 3 of Cav3.2 KO and positive control brain) (Suppl. Fig. S4a). This

Western blot results also confirmed that the KO of Ca$_V$3.2 does not affect its counterpart's isoform Ca$_V$3.1.

Whereas, only two of the clones: clone 3 and 4 exhibited the complete loss of Ca$_V$3.2 protein as depicted in Western blot for Ca$_V$3.2. The remaining clones show faint bands compared to the control (WT control, Ca$_V$3.1 KO and positive control HEK overexpressing Ca$_V$3.2) (Suppl. Fig. S4b). Unlike Ca$_V$3.1 Western blot, Ca$_V$3.2 Western blot showed a faint band of Ca$_V$3.1 clone 2. It is opposite to that of our sequencing result. In sequencing, the clone 2 of Ca$_V$3.1 amplified for Ca$_V$3.2 (Suppl. Fig. S3a-b) exhibited no base deletion and is similar to control. The reason for this is unknown. These results confirmed that CRISPR-cas9-Ca$_V$3.1/ Ca$_V$3.2 transfection with single-cell clonal selection allowed us to obtain cells with Ca$_V$3.1 and Ca$_V$3.2 KO.

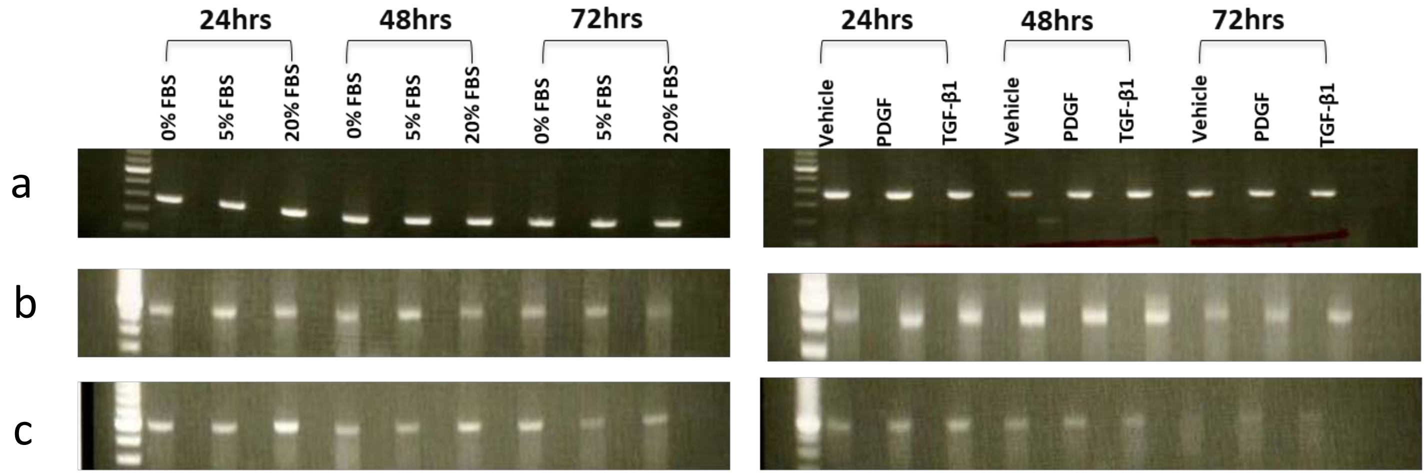

Supplementary Figure S1. Confirmation of TTCC isoform in mouse mesangial cells and modulation of its proliferation by TTCC blockers.

(a, b, c) RT-PCR for the T type calcium isoforms were detected in mouse mesangial cells (MES13). The band in each lane in the agarose gel represent the PCR product of the T type calcium isoforms Cacna1G, Cacna1H and Cacna1I (PCR product size: CaV3.1-376; CaV3.2-594; CaV3.3-528). mRNA of the CaV3.3 in 72 h treatment with PDGF and TGFβ1 alone shown faint band.

a

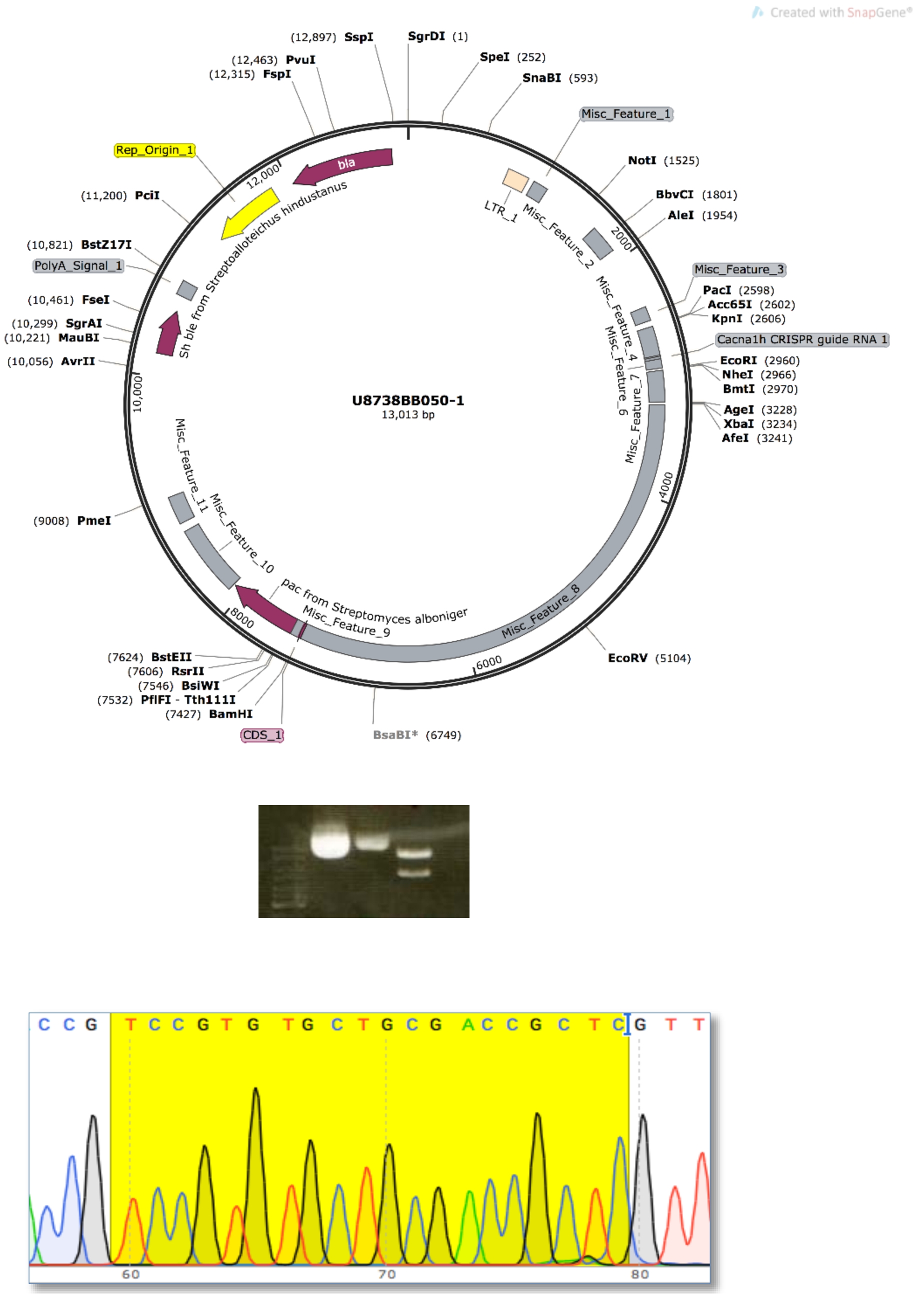
Created with SnapGene®
U8738BB050-1
13,013 bp
(12,897) SspI
SgrDI (1)
SpeI (252)
SnaBI (593)
(12,463) PvuI
(12,315) FspI
Misc_Feature_1
Rep_Origin_1
NotI (1525)
BbvCI (1801)
AleI (1954)
(11,200) PciI
(10,821) BstZ17I
PolyA_Signal_1
(10,461) FseI
(10,299) SgrAI
(10,221) MauBI
(10,056) AvrII
Misc_Feature_3
PacI (2598)
Acc65I (2602)
KpnI (2606)
Cacna1h CRISPR guide RNA 1
EcoRI (2960)
NheI (2966)
BmtI (2970)
AgeI (3228)
XbaI (3234)
AfeI (3241)
(9008) PmeI
EcoRV (5104)
(7624) BstEII
(7606) RsrII
(7546) BsiWI
(7532) PflFI - Tth111I
(7427) BamHI
CDS_1
BsaBI* (6749)
bla
Sh ble from Streptoalloteichus hindustanus
LTR_1
Misc_Feature_2
Misc_Feature_4
Misc_Feature_6
Misc_Feature_7
Misc_Feature_8
Misc_Feature_9
Misc_Feature_10
Misc_Feature_11
pac from Streptomyces alboniger
2000
4000
6000
8000
10,000
12,000
C C G T C C G T G T G C T G C G A C C G C T C G T T
60
70
80

b

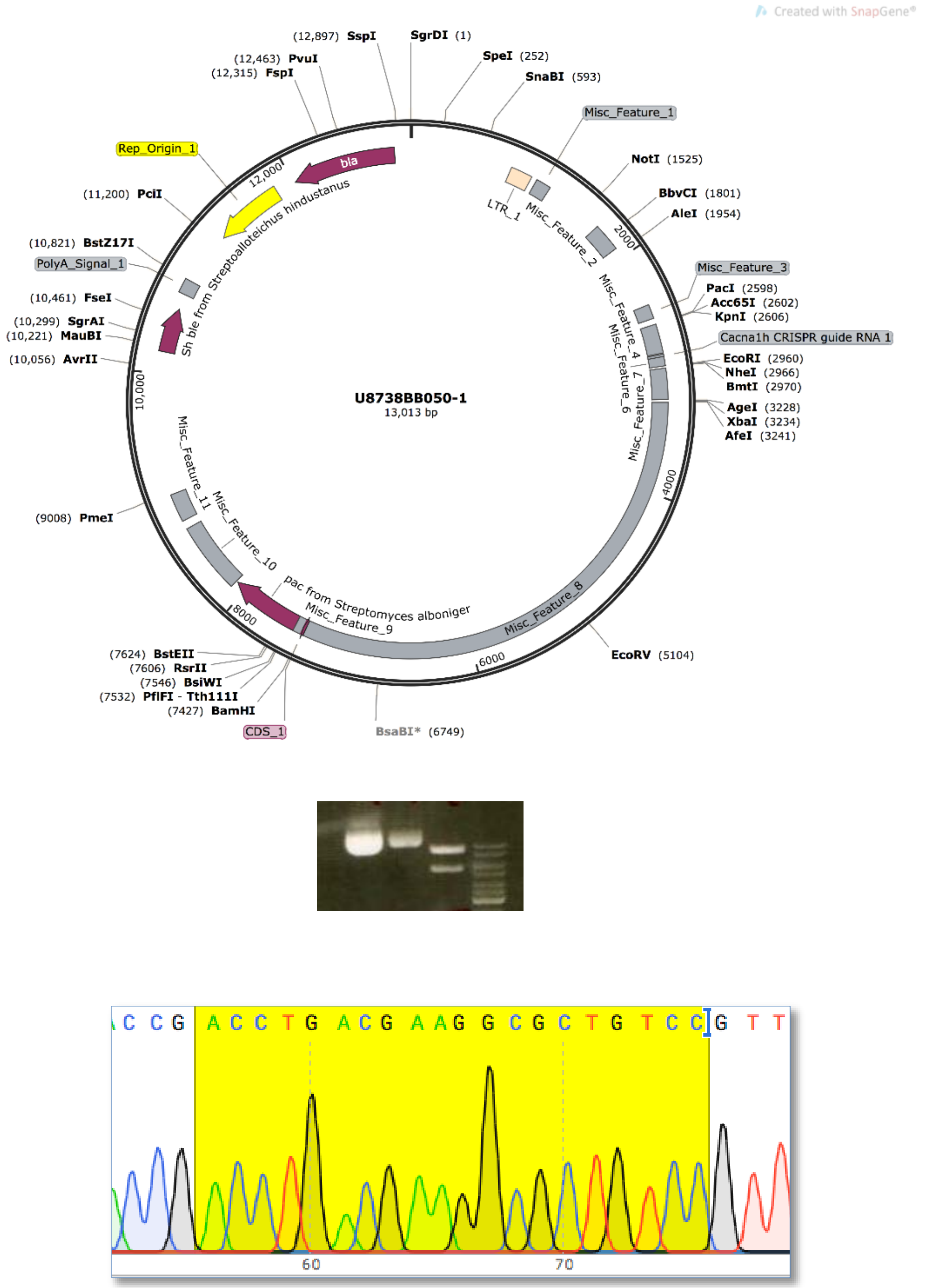
Created with SnapGene®
U8738BB050-1
13,013 bp
(12,897) SspI
SgrDI (1)
SpeI (252)
SnaBI (593)
(12,463) PvuI
(12,315) FspI
Misc_Feature_1
Rep_Origin_1
NotI (1525)
BbvCI (1801)
AleI (1954)
(11,200) PciI
(10,821) BstZ17I
PolyA_Signal_1
(10,461) FseI
(10,299) SgrAI
(10,221) MauBI
(10,056) AvrII
Misc_Feature_3
PacI (2598)
Acc65I (2602)
KpnI (2606)
Cacna1h CRISPR guide RNA 1
EcoRI (2960)
NheI (2966)
BmtI (2970)
AgeI (3228)
XbaI (3234)
AfeI (3241)
(9008) PmeI
EcoRV (5104)
(7624) BstEII
(7606) RsrII
(7546) BsiWI
(7532) PflFI - Tth111I
(7427) BamHI
CDS_1
BsaBI* (6749)
bla
Sh ble from Streptoalloteichus hindustanus
LTR_1
Misc_Feature_2
Misc_Feature_4
Misc_Feature_6
Misc_Feature_7
Misc_Feature_8
Misc_Feature_9
Misc_Feature_10
Misc_Feature_11
pac from Streptomyces alboniger
2000
4000
6000
8000
10,000
12,000
C C G A C C T G A C G A A G G C G C T G T C C G T T
60
70

Supplementary Figure S2. Validation of CRISPR-cas9- gRNA for cacna1G and Cacna1H.

plenti-CRISPR-cas9-cacna1G a) and plenti-CRISPR-cas9-cacna1H b) vector map expressing gRNA mapping to exon 4 of 36 exons containing cacna1G gene and on exon 6 of 35 exons containing cacna1H gene under the control of U6 promoter, with selection markers ampicillin for prokaryotic and puromycin for mammalian cell selection. a) and b) plasmids were confirmed by restriction digestion by MluI restriction enzyme and by Sanger sequencing using primer designed for U6 promoter. (gRNA sequences: Cacnca1G-TCCGTGTGCTGCGACCGCTC, Cacna1H- ACCTGACGAA GGCGCTGTCC).

Gel: Panel A: Lane 1-10KB ladder, Lane 2 –circular plasmid, Lane 3-No restriction enzyme control, Lane 4- plasmid digested by Mlu. Panel B: Lane 1 –circular plasmid, Lane 2-No restriction enzyme control, Lane 3- plasmid digested by Mlu, Lane 4-10KB ladder. Yellow highlight - gRNA sequence.

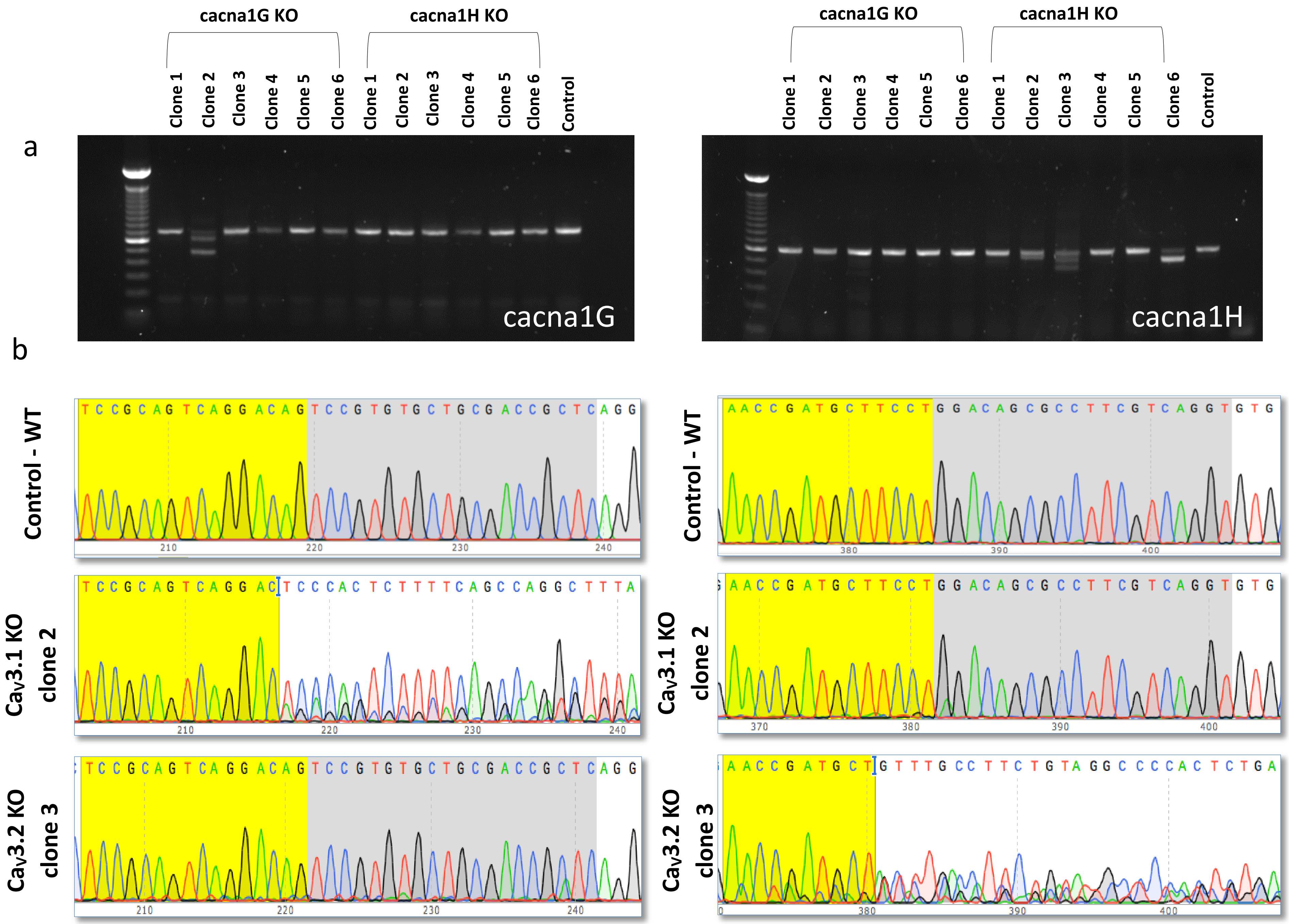
a
cacna1G KO
cacna1H KO
Clone 1
Clone 2
Clone 3
Clone 4
Clone 5
Clone 6
Control
cacna1G
cacna1H
b
Control - WT
Cav3.1 KO clone 2
Cav3.2 KO clone 3

C

Ca_V3.1 and Ca_V3.2 DKO clone 1

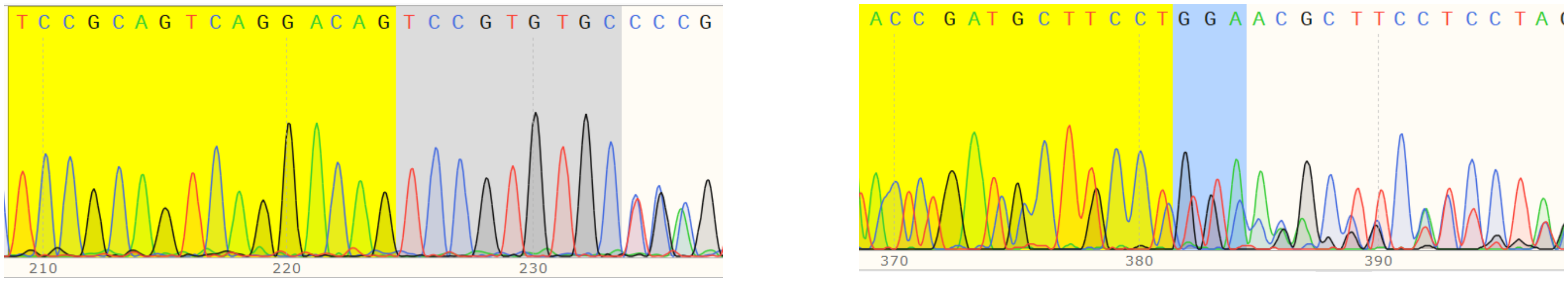

Supplementary Figure S3. Confirmation of Ca_v3.1 and Ca_v3.2 knockout at the genomic level.

a) PCR amplification for Ca_v3.1 (left panel) and Ca_v3.2 (right panel) genes in the six clones each from cacna1G and cacna1h by using primers spanning gRNA region. Cacna1G clone 2 and Cacna1H clone 3 harbouring indel mutation shows PCR product shift compare to control. b) Sequencing analysis of cacna1G and cacna1H SKO in the clone 2 of cacna1G, clone 3 of cacna1H. Sequencing results show deletion of the gRNA sequence with the frameshift mutation compare to wild type (WT) control, confirming the knockouts. c) Sequencing analysis of DKO shows frameshift mutation compare WT as in b). Right panel-sequencing for cacna1G; left panel- sequencing for cacna1H. Yellow highlight represents the sequence before gRNA in the genome and grey highlight represent the gRNA sequence.

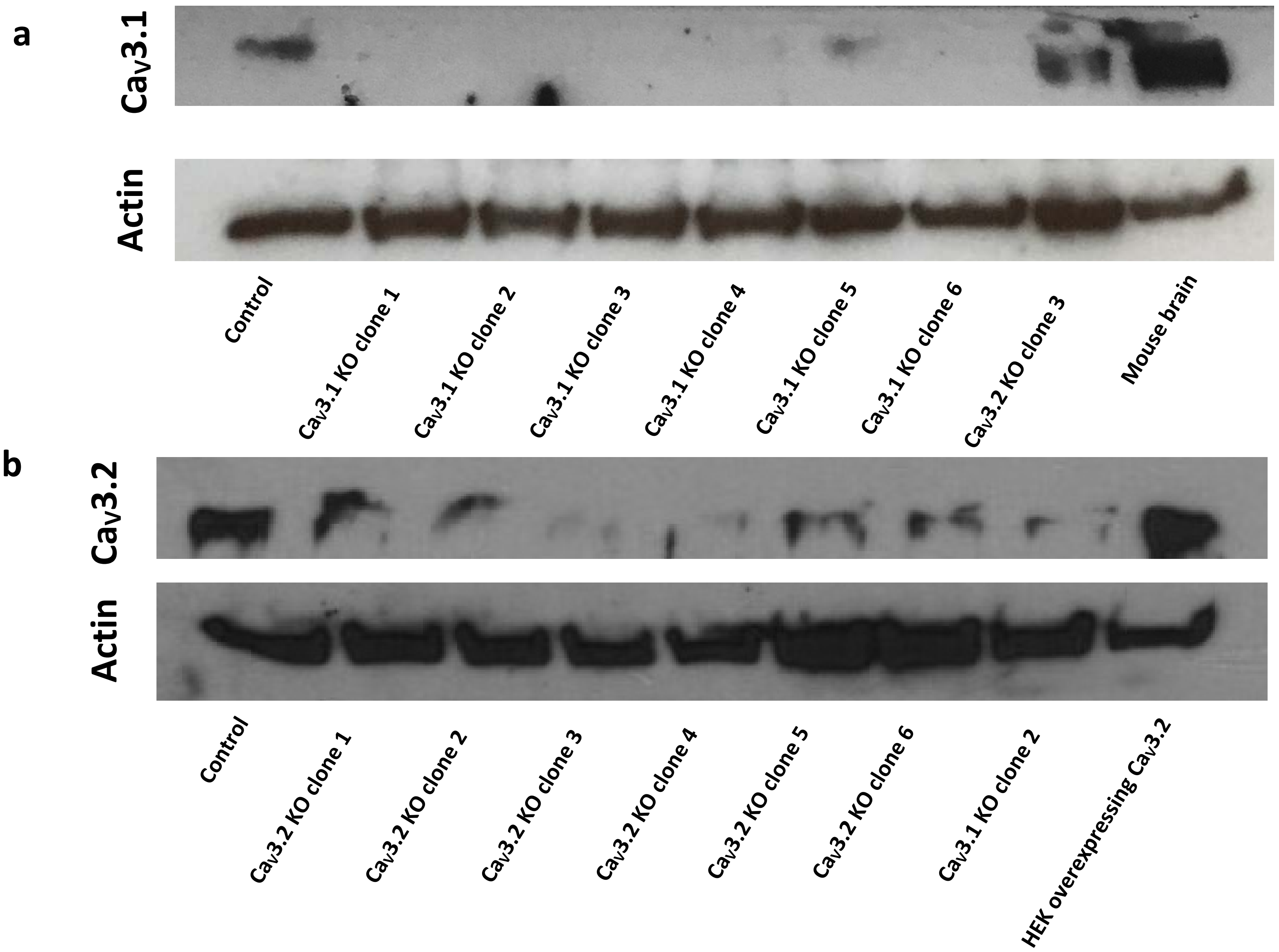
a
Cav3.1
Actin
Control
Cav3.1 KO clone 1
Cav3.1 KO clone 2
Cav3.1 KO clone 3
Cav3.1 KO clone 4
Cav3.1 KO clone 5
Cav3.1 KO clone 6
Cav3.2 KO clone 3
Mouse brain
b
Cav3.2
Actin
Control
Cav3.2 KO clone 1
Cav3.2 KO clone 2
Cav3.2 KO clone 3
Cav3.2 KO clone 4
Cav3.2 KO clone 5
Cav3.2 KO clone 6
Cav3.1 KO clone 2
HEK overexpressing Cav3.2

Supplementary Figure S4. Confirmation of cacna1G and cacna1H knockout at the post-translational level.

Western blot for $Ca_V3.1$ (a) protein expression in the single-cell clones of $Ca_V3.1$ knockout show absence of bands in all six $Ca_V3.1$ KO clones compared to wild type cells and the positive control mouse brain. (b) single-cell clone of $Ca_V3.2$ KO shows the absence of bands in 5 clones compared to wild type and positive control HEK cell overexpressing $Ca_V3.2$. Loading control- actin.

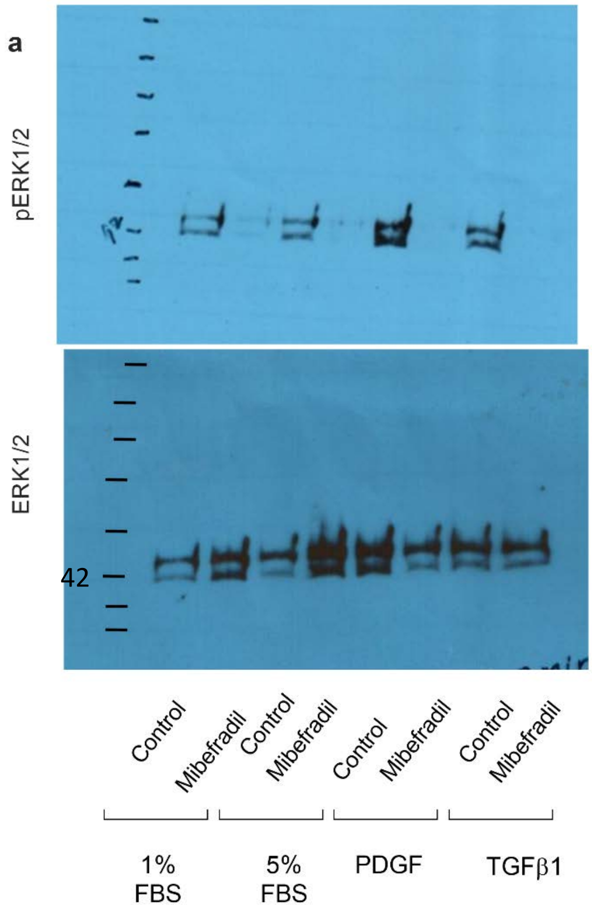

Supplementary Figure S5: Figure shows the full-length image of Western blot for Figure 2a described in the main text.

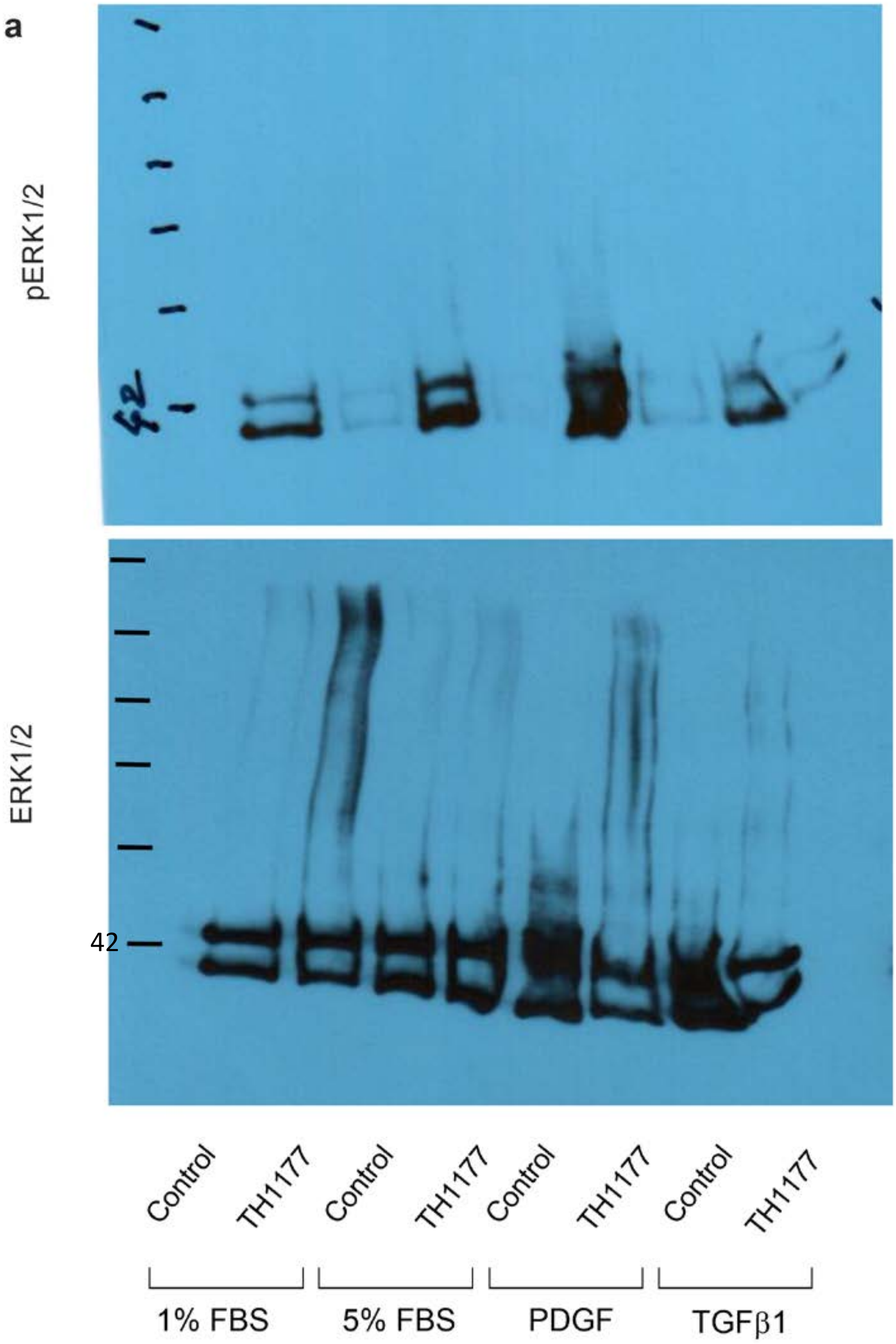

Supplementary Figure S6: Figure shows the full length image of Western blot for Figure 3a described in the main text.

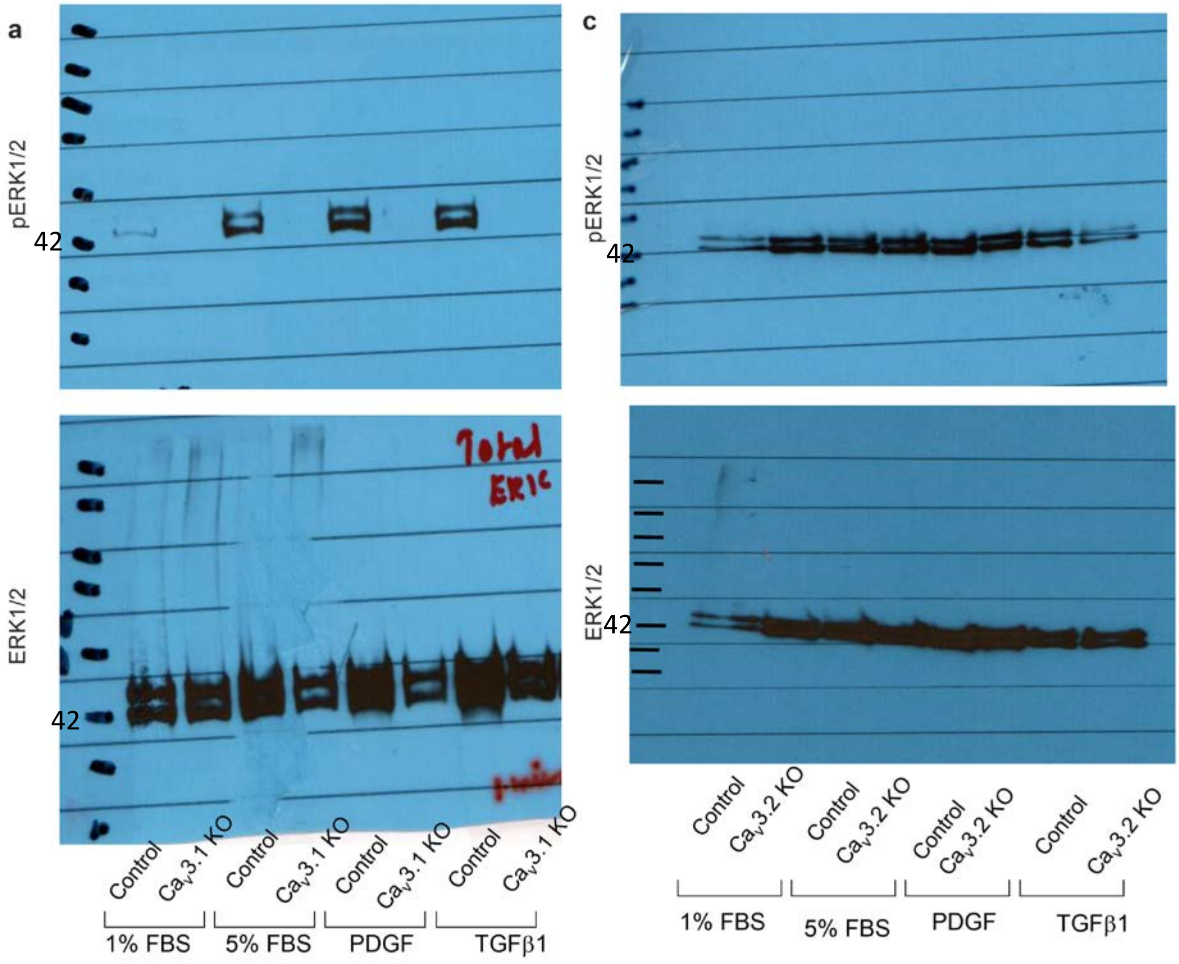

Supplementary Figure S7: Figure shows the full length image of Western blot for Figure 4a and 4c described in the main text.

Supplementary Table S1: Primers list

Primer	Forward	Reverse
	**RT-PCR**	
$Ca_V3.1$	TTGCTGGGATGCTGGAGTAC	GGCACGCTCCTGCAGGAT
$Ca_V3.2$	GAAGATAGCTGCTGCTTCCG	AGTCTCCACGACCAGCTTGA
$Ca_V3.3$	AGCACTGAGCGCATCTTCCT	CACAGCAACAGCATCCAATC
	**PCR**	
$Ca_V3.1$	GGAGACTGTCATTGCTTGGT	ACTGCAAGAGTTGGAAAGGG
$Ca_V3.2$	CAGAAGCTGCTCTGACCTTG	ACAGTCTTAGAGGGAGGGTC
